# Supplementary material for: Maternal information-seeking on pregnancy-induced hypertension and associated factors among pregnant women, in low resource country, A cross-sectional study design
Source: PLOS Digit Health. 2025 Mar 18;4(3):e0000740. doi: 10.1371/journal.pdig.0000740 (PMC11957557; doi:10.1371/journal.pdig.0000740)
Supplement: Attachment — Submitted filename: Recommendations PDIG-D-24-00137.pdf [file pdig.0000740.s002.pdf]

The paper explores the impact of digital health interventions on managing Pregnancy-Induced Hypertension (PIH) in rural Ethiopia. To strengthen its scientific impact, consider addressing the following aspects:

1. Originality and Novelty:
    - Clearly highlight the novel aspects of your research and how it differs from existing studies. A comparative analysis with recent similar research can help establish the unique contributions of your work.
2. Importance and Broad Interest:
    - Emphasize the broader implications of your findings. Articulate how your study influences practice, policy, or further research in digital health, and how it could be relevant beyond the specific context of rural Ethiopia.
3. Methodological Rigor and Ethical Standards:
    - Provide a more detailed description of your study design, data collection methods, and analysis techniques. Ensure all ethical considerations are thoroughly addressed, including obtaining necessary approvals.
4. Substantial Evidence:
    - Include additional analyses or data to further substantiate your conclusions. Address any study limitations openly and discuss how future research could address these limitations.
5. Utility and Accessibility:
    - Elaborate on the practical applications of your findings. Ensure that the implications for practice and policy are clear and accessible to a wide audience, including practitioners and policymakers.
6. Open Science Standards:
    - Deposit your data and related protocols in public repositories to enhance transparency and reproducibility. Provide links or access information in your manuscript.
7. Reproducibility and Protocols:
    - Detail your methodological protocols to facilitate replication of your study. If there were deviations from standard practices, explain and justify these deviations clearly.
8. Clear and Accessible Writing:
    - Revise the manuscript to improve clarity and coherence. Ensure that complex concepts are explained in an accessible manner for non-specialists.
9. Supporting Information:
    - Consider including detailed protocols, algorithms, or additional data as supplementary materials. This can enhance the transparency and depth of your research.
10. Compliance with Guidelines:

- Confirm and mention adherence to relevant guidelines (e.g., CONSORT, STROBE) to ensure the methodological rigor of your study.

Overall, the paper is not yet fully aligned with the publication standards for PLOS Digital Health. The research lacks a clear emphasis on its novelty and unique contributions, making it difficult to discern how it differentiates itself from existing studies in the field. The evidence provided to support the conclusions is insufficient, as the analysis and data are not comprehensive enough to firmly substantiate the claims made. The methods section needs greater detail to ensure reproducibility, which is a crucial aspect of scientific rigor.

Ethical considerations are underrepresented, with insufficient discussion of approvals and ethical guidelines pertinent to the study. Additionally, the practical utility of the findings is not well articulated, leaving the potential applications and implications unclear. The manuscript also falls short in adhering to open science practices, as there is a lack of publicly accessible data and detailed protocols.

Furthermore, the clarity of the writing requires improvement to ensure the manuscript is accessible to a broader, non-specialist audience. Essential supporting materials, such as detailed protocols or algorithms, are missing, which hinders the transparency of the research. Lastly, the study does not reference adherence to relevant research guidelines like CONSORT or STROBE, impacting its methodological rigor and alignment with established research standards.
