# Supplementary material for: Maternal information-seeking on pregnancy-induced hypertension and associated factors among pregnant women, in low resource country, A cross-sectional study design
Source: PLOS Digit Health. 2025 Mar 18;4(3):e0000740. doi: 10.1371/journal.pdig.0000740 (PMC11957557; doi:10.1371/journal.pdig.0000740)
Supplement: Attachment — Submitted filename: PDIG_D_24_00137R1.pdf [file pdig.0000740.s007.pdf]

Reviewer's Report for "Maternal Information-Seeking on Pregnancy-Induced Hypertension and Associated Factors among Pregnant Women in a Low-Resource Country: A Cross-Sectional Study"

November 18, 2024

1 Summary

This manuscript addresses the critical challenge of pregnancy-induced hypertension (PIH), a significant medical condition affecting 6–10% of pregnancies globally and posing severe risks to maternal health. The lack of information-seeking behavior regarding PIH among expectant mothers exacerbates the likelihood of complications and maternal mortality. This study investigates the information-seeking behaviors related to PIH and associated factors among pregnant women in the rural Sekela district. The findings reveal that PIH-related information-seeking is generally low, with healthcare providers identified as the primary source of information. Factors influencing information-seeking include maternal age, family resistance, satisfaction with healthcare services, and perceived severity of PIH.

2 Comments

- **Proofreading:** The manuscript requires thorough proofreading, particularly in the introduction and conclusion sections. While these sections provide a good overview, the lack of clarity and grammatical issues may hinder comprehension from a general reader's perspective.
- **Visualization of Data:** The manuscript includes several tables. To enhance clarity and reader engagement, consider representing the data as percentage-stacked bar plots or other suitable visualizations for better interpretation.

- **Clarity in Methodology:** The methods section needs clearer explanations, particularly regarding the binary and multivariable logistic regression techniques used in the analysis. The current description may be difficult for readers to follow.

3 Overall Recommendation

I recommend that the paper be revised to address the aforementioned issues before further consideration for publication.
