# Supplementary material for: Maternal information-seeking on pregnancy-induced hypertension and associated factors among pregnant women, in low resource country, A cross-sectional study design
Source: PLOS Digit Health. 2025 Mar 18;4(3):e0000740. doi: 10.1371/journal.pdig.0000740 (PMC11957557; doi:10.1371/journal.pdig.0000740)
Supplement: Attachment — Submitted filename: PDIG-D-24-00137_edits.docx [file pdig.0000740.s003.docx]

PLOS Digital Health

Maternal Information-Seeking on Pregnancy-Induced Hypertension and Associated Factors among Pregnant Women, in Low resource country, A cross sectional study design

--Manuscript Draft--

| Manuscript Number: | PDIG-D-24-00137 |
| --- | --- |
| Article Type: | Research Article |
| Full Title: | Maternal Information-Seeking on Pregnancy-Induced Hypertension and Associated Factors among Pregnant Women, in Low resource country, A cross sectional study design |
| Short Title: | Information-Seeking on Pregnancy Induced Hypertension and determinants at Pregnant Women |
| Corresponding Author: | Ayana Muluneh Alebachew, MPH  Wollo University  Dessie, ETHIOPIA |
| Order of Authors: | Ayana Muluneh Alebachew, MPH |
|  | Kegnie Shitu |
|  | Ayenew Sisay |
|  | Sefefe Birhanu |
|  | Adamu Ambachew |
|  | Agmasie Damitew |
|  | Mulugeta Desalegn Kasaye |
|  | Fekade Demeke Bayou |
| Keywords: | - PIH; health information Seeking behavior; pregnant women; rural area; information seeking |
| Abstract: | Introduction  Pregnancy-induced hypertension (PIH) is the most prevalent medical problem associated with pregnancy. It has been reported to affect 6–10% of all pregnant women worldwide. A mother's failure to seek information related to PIH would increase the risk of dying from its complications.  Objective  This study aimed to assess PIH information-seeking behavior and its associated factors among pregnant women in rural Sekela district.  Method  A Community-based cross-sectional quantitative study was conducted among pregnant women from May 15 to June 15, 2022. An interviewer administrated structured questionnaire was used to collect the data. It was calculated using the single population proportion formula using design effect, 10% non-response rate. A cluster sampling technique was used to select sampled in the study area. We conducted a descriptive analysis, bi-variable analysis and multi-variable analysis to identify determinants of PIH information seeking.  Result  The proportion of PIH information seeking among pregnant women was 214 (35.43%). Pregnant mother’s age of 35 years and above (AOR =0.67, 95% CI =.46, .97),  resistance of family (AOR = 0.45, 95% CI=.29, .69), health care satisfaction (AOR=1.7,  95% CI=1.1, 2.5), and perceived severity of PIH (AOR=1.6, 95% CI=1.1, 2.4) were |

|  | significantly associated with PIH information seeking.  Conclusion  According to our finding Information seeking related to PIH founds to be low. The major source of PIH related information was health care providers. Aged mothers, family resistance, mother’s satisfaction on health care service, perceived severity of PIH were found to be associated with PIH information seeking. |
| --- | --- |
| Additional Information: |  |
| Question | Response |
| Government Employee  Are you or any of the contributing authors an employee of the United States government?  Manuscripts authored by one or more US Government employees are not copyrighted, but are licensed under a [CC0 Public Domain Dedication,](https://creativecommons.org/about/cc0) which allows unlimited distribution and reuse of the article for any lawful purpose. This is a legal requirement for US Government employees.  This will be typeset if the manuscript is accepted for publication. | No - No authors are employees of the U.S. government. |
| Financial Disclosure  Enter a financial disclosure statement that describes the sources of funding for the work included in this submission. Review the [submission guidelines](http://journals.plos.org/digitalhealth/s/submission-guidelines#loc-financial-disclosure-statement) for detailed requirements. View published research articles from [PLOS Digital Health](https://journals.plos.org/digitalhealth/) for specific examples.  This statement is required for submission and will appear in the published article if the submission is accepted. Please make sure it is accurate. | The author(s) received no specific funding for this work. |

| Funded studies Enter a statement with the following details: - Initials of the authors who received each award - Grant numbers awarded to each author - The full name of each funder - URL of each funder website - Did the sponsors or funders play any role in the study design, data collection and analysis, decision to publish, or preparation of the manuscript? |  |
| --- | --- |
| Did you receive funding for this work? |  |
| Competing Interests  On behalf of all authors, disclose any competing interests that could be perceived to bias this work.  This statement will be typeset if the manuscript is accepted for publication.  Review the instructions link below and PLOS Digital Health's [competing interests](https://journals.plos.org/digitalhealth/s/competing-interests#loc-what-represents-a-competing-interest) policy to determine what information must be disclosed at submission. | No-Competing Interests among authors |
| Data Availability  Provide a Data Availability Statement in the box below. This statement should detail where the data used in this submission can be accessed. This statement will be typeset if the manuscript is accepted for publication.  Before publication, authors are required to make all data underlying their findings fully available, without restriction. Review our [PLOS Data Policy](https://journals.plos.org/digitalhealth/s/data-availability) page for detailed information on this policy. Instructions for writing your Data Availability statement can be accessed via the Instructions link below. | the data is available from PI-aouthr |

Manuscript	[Click here to access/download;Manuscript;manuscript.docx](https://www2.cloud.editorialmanager.com/pdig/download.aspx?id=45599&guid=7e20078b-efb0-45ed-9eb2-c1138deb7b4d&scheme=1)

1 Maternal Information-Seeking on Pregnancy-Induced Hypertension
2 and Associated Factors among Pregnant Women, in Low resource
3 country**, A** cross sectional study design
4 Ayana Alebachew Muluneh^1*^, Kegnie Shitu^2^, Ayenew Sisay ^3^, Sefefe Birhanu^3^, Adamu
5 Ambachew ^4^	Agmasie Damitew^5^, Mulugeta Desalegn Kasaye^1^	Fekade Demeke Bayou^6^	6	**Affiliations**

7	^1*^Department of Health Informatics, School of Public Health, Wollo University, Dessie, 8	Ethiopia

9 ^2^ Department of Health education and behavioral science, Institute of public Health, 10 University of Gondar, Gondar, Ethiopia

11 ^3^Department of Health Informatics, College of Medicine and Health Sciences, Debremarkos 12 University, Debremarkos. Ethiopia

13 ^4^Department of Health Informatics, College of Medicine and Health Science, Mettu 14 University, Dessie, Ethiopia

15	^5^Department of Health Informatics, School of Public Health, Debre Birhan University, Debre 16	Birhan, Ethiopia

17	^6^Department of Epidemiology and Biostatistics, School of Public Health, Wollo University, 18	Dessie, Ethiopia

19

20 **Corresponding author***
21 Ayana Alebachew Muluneh
22 Email: alayu382@gmail.com
23 Mobile: +251 929269638

# Abstract

**Introduction: -** Pregnancy-induced hypertension (PIH) is the most prevalent medical problem associated with pregnancy. It has been reported to affect 6–10% of all pregnant women worldwide. A mother's failure to seek information related to PIH would increase the risk of dying from its complications.

**Objective: -** This study aimed to assess PIH information-seeking behavior and its associated factors among pregnant women in rural Sekela district**.**

**Method: -** A Community-based cross-sectional quantitative study was conducted among pregnant women from May 15 to June 15, 2022. An interviewer administrated structured questionnaire was used to collect the data. It was calculated using the single population proportion formula using design effect, 10% non-response rate. A cluster sampling technique was used to select sampled in the study area. We conducted a descriptive analysis, bi-variable analysis and multi-variable analysis to identify determinants of PIH information seeking.

**Result: -** The proportion of PIH information seeking among pregnant women was 214

(35.43%). Pregnant mother’s age of 35 years and above (AOR =0.67, 95% CI =.46, .97), resistance of family (AOR = 0.45, 95% CI=.29, .69), health care satisfaction (AOR=1.7, 95% CI=1.1, 2.5), and perceived severity of PIH (AOR=1.6, 95% CI=1.1, 2.4) were significantly associated with PIH information seeking.

**Conclusion**: - According to our finding Information seeking related to PIH founds to be low. The major source of PIH related information was health care providers. Aged mothers,

44 family resistance, mother’s satisfaction on health care service, perceived severity of PIH 45 were found to be associated with PIH information seeking.

46 **Key words**: - PIH, health information Seeking behavior, pregnant women, rural area,
47 information	seeking

## Introduction

Pregnancy-induced hypertension (PIH) is defined as new hypertension that appears at 20 weeks or more gestational age of pregnancy with or without proteinuria, which includes gestational hypertension, preeclampsia, and eclampsia(1, 2). Hypertensive disorders of pregnancy are the major complications that cause about 60%–80% of all maternal deaths. Preeclampsia is a major hypertensive disorder of pregnancy that has caused maternal mortalities and morbidities all around the world (3). PIH is a significant public health warning and cause of neonatal morbidity and mortality, especially in developing countries, accounting for more than 10 million LBW infants, and women who had PIH were nearly ten times at increased risk of LBW

(4).

Pregnancy-induced hypertension (PIH) information-seeking behavior is regularly vital for the advancement of knowledge, behavioral changes, health decisions, and the sharing of PIH health information among women (5). Currently, health information seeking behavior (HISB) is broadly viewed as the ways by which individuals obtain information about health, illness, health promotion, and risks (6). Information-seeking behavior (HISB) is a form of self-fulfillment involving the gathering of information about health from a variety of sources (7). Information on health that leads to psychosocial adjustment to the known risk is crucial for health promotion (8). Health information seeking is used for the prevention, control, and psychological arrangement of individuals for the occurrence of risk or disease, and this is not only for the occurrence of risk or disease but also for life management (9). Maternal health concerns account for a large share of women's health problems. For starters, pregnancy-induced hypertension diseases are a primary source of maternal morbidity and mortality globally, affecting around 5–10% of pregnant women, or 8 million (10). A study conducted at Hindu University states that pregnancy-induced hypertension is the leading cause of maternal and neonatal mortality, and 60.9% of pregnant women had less information on pregnancy-induced hypertension. This is why the reason for poor control of hypertension in these women might be limited PIH self-care knowledge(11).

Most pregnant mothers are not confident in seeking information from health care providers related to pregnancy-induced hypertension; this is why most pregnant mothers say they have no faith in themselves (12). During pregnancy, there is so much information that women need to be fed, like the place of delivery, pregnancy period, nutrition, miscarriage, pregnancy complications and danger signs, and medicines in pregnancy (13).

Pregnant mothers delay seeking information on PIH, increasing their risk of dying from complications (14). It is also estimated that pregnancy-induced hypertension (PIH) is one of the hypertensive disorders of pregnancy that affects about 6–10% of all pregnant women worldwide (15). Information-seeking behavior is dynamic and non-linear and's skilled as a method of interaction of thoughts, emotions, and action. It is linked to plenty of interpersonal conversation behaviors beyond asking questions to include techniques such as the applicant's answer (16).

Low health literacy has been linked to poor understanding and usage of health information, inadequate preventive behaviors, unsafe self-care, chronic disease management, and increased medical costs. It has recently become one of the health care priorities in more developed socioeconomic countries (17). Low Maternal Health Literacy(MHL) is one of the various leading causes, which also include social and demographic problems, incomplete accessibility, and poor maternal and child health service quality (18).

Health literacy may influence an individual’s information seeking and subsequently her/his health knowledge and health behavior. The Study shows that the relationship between health literacy and information seeking in pregnant women (17).

Information seeking is a process by which a person purposefully needs to change the status of their knowledge. It is interesting for human beings to predict something for the future. People are seeking to address their information needs. People use the information to solve problems, do work, or increase their knowledge. So, identifying their seeking behavior as a part of social behavior helps us build better information systems (3). In the world, 10% of pregnant women are complicated by hypertension, with preeclampsia and eclampsia being the major causes of maternal and perinatal morbidity and mortality (12). Pregnancy-induced hypertension is the most prevalent medical problem associated with pregnancy, affecting 6–10% of all pregnancies worldwide. It is the world's second-leading cause of maternal and fetal death. This is caused by low health information seeking (15, 19).

The World Health Organization estimates that at least one woman dies every seven minutes from complications of hypertensive disorders during pregnancy (15). The study conducted in the Tigray region shows that the prevalence of pregnancy-induced hypertension in Ethiopia is 2.2% to 18.3%. This indicates that there is low awareness or information about pregnancy-induced hypertension prevention or control mechanisms (20). The study conducted in the USA states that women with hypertensive pregnancy disorders should have a comprehensive plan of care, which includes prenatal counseling, frequent visits during pregnancy, timely delivery, appropriate intrapartum monitoring and care, and postpartum follow-up (21).

The guideline for the management of hypertension before, during, and after pregnancy states that treatment is mandatory for severe hypertension (22). The study conducted in India states that in developing countries, women have little or no information on the types of pregnancy-induced complications or where to go for complications (23). The systematic review conducted in Ethiopia shows poor access to health care, distance from health facilities, and level of education are the main side effects of maternal and infant mortality (24). PIH disease is the amount of salt in the diet, alcohol consumption during pregnancy, increasing the number of fried foods and junk food you eat, irregularly exercising, and did not get enough rest (11). According to a systematic review conducted in Ethiopia, 75% of maternal deaths are caused by known cases and preventable diseases such as high blood pressure during pregnancy, complications during delivery, unsafe abortion, severe bleeding, and infections during and after birth (24). The study conducted in rural Tanzania shows that telecenters, libraries, and other health information providers' services are gender-sensitive to facilitate equitable access to relevant information for women for rational decision-making (25, 26).

**Methods**

A community-based cross-sectional quantitative study design was conducted in rural Sekela woreda. This study was conducted in rural Sekela woreda, northwest Ethiopia. Sekela is found 165 kilometers south of Bahir Dar. Gish Abay is a town in Sekela woreda, located 430 km from Addis Ababa, Ethiopia. Based on the 2007 national census conducted by the Central Statistical Agency of Ethiopia (CSA), the Sekela woreda has a total population of 138,691, an increase of 61.36% over the 1994 census, of whom 69,018 were men and 69,673 were women; 6,779, or 4.89%, are urban inhabitants.

**Study period**

This study was conducted from May 15 to jun15 /2022 in rural Sekela woreda northwest Ethiopia.

**Source population**

The source population was pregnant women who had lived in rural Sekela Woreda.

**Study population**

The study population was pregnant mothers in rural Sekela Woreda.

**Inclusion criteria**

The inclusion criteria were pregnant women who had lived in rural Sekela Woreda for at least six months.

**Exclusion criteria**

Pregnant women who were admitted for delivery services and temporary residents visited the study area (rural

Sekela woreda were excluded from the study.

Sample Size and Sampling Procedures

Due to the absence of a prior study in the similar country, we used 50% prevalence to determine the sample size.

The sample size of this study was calculated using a single population proportion formula.

Where: - n= the required sample size. Z = the value of standard normal distribution corresponding

144 to α/2= 1.96. p = predicted proportion of PIH Information-seeking behavior of pregnant women. q = 1-p 145 predicted proportion of PIH Information-seeking behavior of pregnant women. d= the margin of error 5 %( 0.05).

146 But, we could not find any study about the PIH information-seeking behavior of pregnant women that determines 147 the HISB of pregnant women. So, for the single population proportion, we took proportion (p) 50%.

Due to the sampling stage, we were using Design Effect 1.5; the last calculated sample size was 635. In our cluster, there are 30 rural Kebles. To select a sample Keble from the cluster, we randomly selected a Kebeles to study. Among these Kebeles, a sample was collected from each Kebeles based on the number of pregnant women they have. We were interviewed in the selected area until the sample was completed. Totally, 14 Kebeles were included in the study sample. The selected pregnant women were interviewed using a structured Amharic language-version questionnaire. During the data collection period, we interviewed all the pregnant women who were in the selected Keble. The total number of pregnant women in these 14 Kebeles was 637.

### Operational definition

Information Seeking Behavior: PIH-ISB is characterized by the PIH information sources, types of PIH information, and trust in PIH information sources (5, 6, 59)

PIH information seeking: - We were measured through a questioner. Whether the pregnant women knew about PIH before or not. Yes or No question. Responses are coded as 0 for NO and 1 for YES, respectively (5, 53).

Use of Information Sources: - We asked participants how often (never, sometimes, often, or all the time) they used different information sources to receive pregnancy-induced hypertension health information in the past 12 months, then identified the most frequently used source for seeking information about PIH by using the mean score. We will identify their information source (5, 47).

Alcohol drinking: The safest choice for a woman who is pregnant or planning to become pregnant is not to drink alcohol. So, if she says “drink alcohol," and if not, she is not drinking alcohol (60, 61).

Distance: Distance: This is the distance from a pregnant mother to the health facility. It was measured as greater than or equal to 60 minutes far and less than 60 minutes not far (24, 50).

Health efficacy: measures how confident the pregnant women are about their aptitude to take good care of their health. It is measured as confident or not confident (5, 53, 59).

**Perceived susceptibility to PIH**: assess the perception of the risk of contracting a PIH. We were measured concerned and not concerned (5, 53).

**Perceived severity: -** Assess the seriousness of contracting a PIH. The respondent’s response was severe, and the respondent’s response was not severe (31, 53).

Data Collection Procedure and Quality assurance

### Data collection tool

A pre-test and structured face-to-face interview (structured question) were used to collect data from study participants. The study’s instrument (tool) was adapted from the health information national survey tool (62) . This is to adapt the PIH information-seeking behavior questionnaire. Different questions measuring sociodemographic characteristics, PIH information sources, behavioral characteristics, technological factors, and environmental factors were included in the data collection tool (8, 12, 53, 59).

## Data collection procedure

The data collection procedure was a face-to-face interview using an interviewer-administrated Amharic version questionnaire in the rural community. The data was collected by nine data collectors who were BSc health informatician and BSc midwifery professionals. Two supervisors (a health informatician and a midwife professional) were recruited to follow up on the procedure for the data collection. The principal investigator controlled all the activities.

## Data Quality Assurance

One day of training was given for data collectors and supervisors on research ethics, providing informed consent, data collection procedures, data collection tools, how to approach participants, data confidentiality, and respondent’s rights, as well as all the study protocols that were followed throughout the data collection period. Continuous monitoring was done by supervisors throughout the data collection period to ensure that the data was collected according to the study protocol. Before the actual data collection, a pretest of the questionnaire was conducted among pregnant women (10%) of the sample.

Before the study period at Megdom Aser Kebele, which is located in Quarite district, nearby Sekela district.

## Data-Measurements

PIH information seeking behavior was measured with different items adapted from the health information national survey tool that determines whether respondents sought PIH information in the past 12 months (47, 63). Subsequently, the participants were asked about the frequency of PIH information seeking and trusted sources of PIH. PIH information-seeking behavior can be characterized by PIH information sources, types of PIH information, and trust in PIH information sources. Tools for alcohol drinking can be measured by pregnant women, whether they drink alcohol or not at all. So, if she says "drink alcohol,” current drinker and if no, she does not drink alcohol (60, 61). distance of health center from pregnant mother can be measured as greater than

60 minutes far and less than 60 minutes not far (24, 50).

### Data processing and analysis

The data entry form was created using Epi-data 4.6 versions, and the analysis was carried out using STATA 14.1. To ascertain factors associated with PIH information-seeking behavior, bi-variable and multivariable logistic regression were used. Descriptive statistics such us frequencies, means and standard deviations were computed to summarize the study results. The bi-variable logistic regression method was used to analyze the association between each independent variable to the dependent variable (PIH information-seeking behavior) and to select candidate variables for the multiple logistic regression analyses. Variables with a p-value of less than or equal to 0.2 in the bi-variable logistic regression was entered into the multi-variable logistic regression model. Finally the multivariable logistic regression model was fitted to identify factors associated with participants’ information seeking behavior about PIH. Global model fitness of the final model was cheeked using the Hosmer and Lemeshow test in which its P-value was 0.9103 indicated the model was good fit, and 95% confidence interval with its corresponding p-value of less than 0.05 was used to declare statistical significance.

### Ethical consideration

The study was conducted after ethical approval was obtained from the institution of Review Board (IRB) of the University of Gondar. In addition to IPH Official permission letter, we were obtained an Official permission letter from Sekela woreda health office, and informed written consent was also seen by each study participant before the actual data collection was started. The participant were informed for the risks and benefits of the study, they have had the right to withdraw anytime, how confidentiality was maintained using codes, and their right to get their results for free.

**Result**

## Socio-demographic characteristics

A total of 604 pregnant women were participated in this study, with a response rate of 95%. Most participants 419(69.4%) were farmers, 67(11.09%) were merchants, 53(8.77%) were employers, 46(7.6%) were housewife and the remaining 30.6% were included by others, like housewives, merchants, and 19(3.15%) were Private business. The mean ages of the participants were 31.8 ± 6.09 SD, with a minimum and a maximum age of 20 and 45 years, respectively. More than half of the mothers 348 (57%) were < 35 years old. The marital status of the participants, 512 (84.77%) were married. More than half of the participants 354 (58.61%) could not read and write, but 41.39% of the participants could read and write. The demographic distribution of participants is presented in (**Error! Reference source not found.**).

Table 1**:** Socio-demographic characteristics of pregnant women in Rural Sekela woreda (N= 604)

Variables	Category	frequency	Percent (%)

Age	>=35 years old	256	42.38

<=34 years old	348	57.6

Marital Statues	Married	512	84.77

Single	43	7.12

Divorced	49	8.11

Educational Statues	Cannot read & write	354	58.61

Can read and write	51	8.44

primary schooling	109	18.05

secondary schooling	37	6.13

diploma and more	53	8.77

maternal occupation	Farmer	419	69.37

Private business	19	3.15

Merchant	67	11.09

Housewife	46	7.62

Employee	53	8.77

## Technological characteristics

Majority of the study participants, 384 (63.58%) did not use mobile-phone . only 220 (36.4%) of the participants were mobile users. 25 (11.36%) of the participant were current mobile internet users. the internet satisfaction level of the participant 16 (64%) were very satisfied and 6(24%) of the participants were satisfied (Table 2). however majority of the study participants were did not use mobile internet for PIH information seeking purpose . Among 25 study participants who used the internet, 10 (40%) were not used for PIH disease related information searching purposes (**Error! Reference source not found.**)

Table 2: Technological factor of PIH information seeking for pregnant women in Rural Sekela woreda north, Ethiopia 2022

Variables	Category	Frequency	Percent (%)

| Phone owner | Yes | 220 | 36.42 |
| --- | --- | --- | --- |
|  | No | 384 | 63.58 |
| internet access currently | Yes | 25 | 11.36 |
|  | No | 195 | 88.64 |
| level of Satisfaction with the internet | Very satisfied | 16 | 64.00 |
|  | Satisfied | 6 | 24.00 |
|  | unsatisfied | 2 | 8.00 |
|  | Very unsatisfied | 1 | 4.00 |
| Use of Internet for PIH information | Yes | 15 | 60.00 |
|  | No | 10 | 40.00 |

## Behavioral and health related characteristics

Among the total of 604 participants, 392 (64.9%) were current alcohol drinkers. Most of the mothers drank Tela and Arekie 212 (53.94%) and 179 (45.55%), respectively. According to our findings, the majority of pregnant women drink Tela on occasion, up to two glasses, and the others drink Arekie and Tela up to three glasses or three melekiya of drunken alcohol per occasion. As the results showed, there were no cigarette smokers among the study participants (Table 3). Of the total participants, 393 (65.07%) were not confident in their health. Among the total, 211 (34.93%) of the respondents were confident about their health on PIH disease prevention. Among the participants, 130 (21.52%) were resisted by their family to access PIH information by their husbands 63 (48.8%) and by their religious fathers 53 (41.09%). Among the study participants, 571 (94.54%) had no PIH signs or symptoms. However, most of the participants did not experience the signs and symptoms, 360 (59.6%) did not feel healthy (**Error! Reference source not found.**).

Table 3: Behavioral characteristics of PIH in pregnant women in Rural Sekela woreda in north Ethiopia, 2022

Variables	Category	Frequency	Percent (%)

| Current drinker | Yes | 392 | 64.90 |
| --- | --- | --- | --- |
|  | No | 212 | 35.10 |
| Types of drinking | Arekie/ Katikal | 179 | 45.55 |

|  | Tela | 212 | 53.94 |
| --- | --- | --- | --- |
|  | Beer | 2 | 0.51 |
| Cigarette smoking | Yes  No | 0 604 | 0.00. 100.00 |
| would you say your health  is safe | confident  Not confident | 211  393 | 34.93  65.07 |
| Family history of PIH | Yes | 194 | 32.12 |
|  | no | 335 | 55.46 |
|  | I’m not sure | 75 | 12.42 |
| PIH signs and symptoms | Yes | 33 | 5.46 |
|  | No | 571 | 94.54 |
| Health statues | feel healthy | 244 | 40.4 |
|  | not feel healthy | 360 | 59.6 |
| Resistance of family | Yes  No | 130  474 | 21.52  78.48 |
| The most protest of Mothers | My husband  My children  My husband's family  My own family | 63  4  4  6 | 48.84  3.10  3.10  4.65 |

Religious Fathers	53	41.09

## Psychological characteristics

Of the total respondents, about 122 (20.20%) were concerned of got PIH for their lifetime, and 482 (79.8%) were not concerned to get PIH disease in their lifetime. However, most of the participants did not hesitate to suspect PIH. 212 (35.1%) agreed that PIH complications are deadly diseases. On the other hand, 392 (64.9%) did not agree that PIH is a deadly disease. Although among the participants, only 208 (34.44%) of them perceived the severity of PIH. As we have seen from the result, the participants were not psychologically ready for the occurrence of pregnancy-induced hypertension complications in their life time. This led to an increase in the prevalence of PIH disease in the study area. One in five (20.2%) pregnant mothers was concerned about the likelihood of having pregnancy-induced hypertension. Approximately one-third (34.4%) and 412 (65.6%) of pregnant women believe PIH is severe and fatal, respectively (**Error! Reference source not found.**).

Table 4: Psychological characteristics of pregnant women in Rural Sekela Woreda. North, Ethiopia 2022

| Variables | Category | Frequency | Percent (%) |
| --- | --- | --- | --- |
| Likely get PIH | concerned | 122 | 20.20 |
|  | not concerned | 482 | 79.80 |
| perceive the severity of PIH | Severe  Not sever | 208  396 | 34.44  65.56 |
| PIH is a deadly disease? | I Agree | 212 | 35.1 |
|  | I’m not Agree | 392 | 64.90 |

## Environmental Characteristics

Of the total 461, (76.32%) of the participants got to the health center after walking for more than a hour. 143 (23.68%) took less than an hour to get health service from the health center. As the result shows that 461(76.3%) of the participants had a health center problem, and when we looked at the health care professional's service satisfaction, 393 (65.07%) of the participants were not satisfied with the care provider’s service, and 211 (34.93%) of the study participants were satisfied with the care provider’s service. When we look over the advice accessed from healthcare providers, 34.6% of the pregnant mothers did get advice from healthcare providers (**Error! Reference source not found.**).

Table 5: Environmental factor for pregnant women for PIH disease in rural Sekela woreda north, Ethiopia, 2022

| Variables | Category | Frequency | Percent (%) |
| --- | --- | --- | --- |
| Distance of health center from home | far | 461 | 76.3 |
|  | Not far | 143 | 23.68 |
| health professional service satisfaction | Yes  No | 211  393 | 34.93  65.07 |
| Care provider advice | Yes i advice | 209 | 34.6 |
|  | I did not advice | 395 | 65.4 |
| Health center problem | Yes  No | 461  143 | 76.3  23.68 |

## Nutritional characteristics

Of the total, 415 (69.28%) of the study participants did not use fruit. On the other hand, 184 (30.73%) were using fruit in their diets. More than half of the participants did not use sugary foods during their pregnancy, as the result shows below (Table 6). The result shows below, of the total 486(80.46%) were non-iodinated salt users and the rest 118(19.54%) were iodinated salt users. Among the total participants, 293(48.8%) were eating vegetables. Among the total participants, 311(51.49%) of the participants were not vegetarian. As the result shows, of the total, 97.3% of the participants used salt all the time during cooking, and only 16(2.65%) of the participants used salt sometimes in their cooking (**Error! Reference source not found.**).

Table 6: nutritional characteristics of pregnant women in rural Sekela woreda north Ethiopia

| Variables | Category | Frequency | Percent (%) |
| --- | --- | --- | --- |
| sugary foods | Yes | 282 | 46.69 |
|  | No | 322 | 53.31 |
| Usage of fruit per week | i did not eat fruit | 415 | 69.28 |
|  | I eat fruit | 184 | 30.73 |
| Types of salt usage | Iodinated | 118 | 19.54 |
|  | Non Iodinated | 486 | 80.46 |
| Frequency of salt usage | Always | 588 | 97.35 |
|  | Sometimes | 16 | 2.65 |
| Usage of vegetable per week | Yes | 293 | 48.51 |
| No | | 311 | 51.49 |

## Frequency of PIH Information seeking

Of the total PIH information sought by participants, 140 (65.47%) of participants sought PIH information once a month and 64 (30.14%) of the participants sought PIH information occasionally. The other was sought by 7(3.39%), 2 (0.79%), and 1(0.19%), and was sought every day, within three months, and once every six months, respectively (**Error! Reference source not found.**).

65

%

30

%

3

%

1

%

1

%

**frequency of seeking PIH information for pregnant women**

**in rural Sekela woreda north, Ethiopia 2022**

**.**

once a month

sought PIH information occasionally

every day

within three months

every six months

**Figure 1: frequency of seeking PIH information for pregnant women in rural Sekela woreda north, Ethiopia 2022**

## For whom the participants sought PIH information

Of the total information sought participants, 190 (88.9%) sought PIH information for themselves, 20 (9.4%) sought PIH information for their family, friends, and the remainder 4 (1.68%) were for family and self (**Error!**

**Reference source not found.**).

306

190

20

4

0

50

100

150

200

for me

for family

and friends

for family and

self

**c**

**o**

**u**

**n**

**t**

**For whom the PIH information is collected**

for me

for family and friends

for family and self

307	**Figure 2: for whom pregnant women sought PIH information rural Sekela woreda north, Ethiopia, 2022**

### 308 **Source of PIH health information for pregnant women**

309 Participants were asked about their sources of health information for PIH disease. As the result shown in the
310 figure below, 124 (58.1%) of participants used healthcare providers as their PIH information source, and
311 67(31.29%) of the participants used family or their friends as a source of PIH information. So, we can say that
312 books, newspapers, radio, and the internet had fewer users for PIH information as a source (**Error! Reference** 313 **source not found.**).

314

315	**Figure 3: Source of health information in PIH disease for pregnant women in rural Sekela woreda north, Ethiopia, 2022**

## Type of PIH Information

Of the total information sought participant 131(61.3%) were sought information for all types of PIH disease. The other participants were for specific PIH disease types they were sought PIH information. As we were saw on the graph below 39 (18.1%), 31(14.5%) and 13(6%) were sought for preeclampsia, gestation and for eclampsia PIH information they were sought.

## Trusted information sources by pregnant women for PIH

Of the study participants, 110 (51.6%) trusted health care providers and 79 (37.1%) trusted their family or friends for their PIH information source. The rest, 12 (5.64%), 7 (3.35%), 4 (1.37%), and 2 (0.91%), respectively, trusted the internet, radio, newspapers and books (**Error! Reference source not found.**).

**Figure 4: Trusted PIH information Source for pregnant women in rural Sekela woreda north, Ethiopia, 2022**

## Reason for not Seeking Information on PIH

Of the total participants who had not sought PIH information, 268(68.79%) of the participants were afraid to seek PIH information. 90 (23.3%) of the participants also believed that seeking PIH information with other people, including care providers, was inappropriate, while 25 (6.2%) believed that PIH information was uninteresting, and the remaining 7 believed that online information was from an untrusted source. see in (**Error! Reference source not found.**)

Table 7: Reasons for not seeking PIH information in rural Sekela woreda north, Ethiopia, 2022

| Reasons |  | Frequency | Per cent (%) |
| --- | --- | --- | --- |
| Fear about becoming difficult with PIH | 268 |  | 68.79 |
| PIH information is perceived as irrelevant | 90 |  | 23.32 |
| PIH information is not interesting | 25 |  | 6.21 |
| Mistrust of online information | 7 |  | 1.68 |

Proportion of information seeking related to Pregnancy Induced Hypertension. Overall, 214(35.4%) of participants were sought PIH information (**Error! Reference source not found.**).

**Figure 5**

**:**

**Proportion of information seeking related to PIH**

35.43

%

64.67

%

0

10

20

30

40

50

60

70

Yes

No

**I**

**npercent**

Proportion of information seeking related to PIH i

n the form of

percent(%)

Information seeker

not information seeker

### Factors associated with PIH information-seeking behavior

Bi-variable and multi-variable logistic regression were undertaken to select significant factors associated with

PIH information seeking behavior. The results in bi-variable logistic regression analysis were included:- SocioDemographic characteristics (Age), Technological characteristics (phone-owner), psychological characteristics

(self-efficacy and Perceive the severity of PIH), Environmental/organizational characteristics (service

Satisfaction and distance), Health related characteristics (is PIH a deadly disease and ANC follow up),

Behavioral characteristics (Fruit consumption and family-resistance) were found to be significantly associated to PIH information seeking Variables with a P-value less than 0.2. In a multivariable analysis, the mother's age, family resistance, perceived severity of PIH, and health care service satisfaction were significantly associated with PIH information-seeking behavior with a P-value less than 0.05 undertook for discussion. The odds of information seeking among age >= 35 years of pregnant mother were 33% (AOR = 0.67, 95% CI = .29, .69) times less likely to seek PIH related information than that of the women whose age is under 35 years. Family resistance of pregnant women is another significant variable for the outcome variable. So, a pregnant women who were resisted by their family, (AOR= 0.45, 95% CI = .26, .63) were 55% times less-likely to sought PIH information than pregnant women who were not resisted by their family to sought PIH information. Regarding to health services satisfaction, pregnant women, who satisfied with health care service were 1.7 (AOR = .59, 95% CI = 1.1, 2.5) times more likely to seek PIH information than that of the pregnant women who were not satisfied with the health service given by health care provider. In addition to this, the severity of PIH was another significant factor for the outcome variable. Pregnant women who knew the severity of PIH were 1.6 (AOR = 1.6, 95% CI = 1.1, 2.4) times more likely to seek PIH information than those participants who did not know the severity of PIH. The other variables were not significantly associated. See in (**Error! Reference source not found.**)

Table 8: Factors associated with PIH information-seeking among pregnant women in Sekela woreda north

Ethiopia, 2022

| Information sought recode | PIH information-seeking  Yes N(%) No N (%) | COR (95%) | AOR (95% ) |
| --- | --- | --- | --- |
| Age of the mother |  |  |  |
| <35 years | 137(39.4) 211(60.6) | 1 | 1 |

| >=35 years 77(30) | | 179(69.9) | .66(.47,.93) | .67 (.46 , .97)* |
| --- | --- | --- | --- | --- |
| Resistance of family for information of PIH | |  |  |  |
| No | 152(32.1) | 322 (67.9) | 1 | 1 |
| Yes | 62(47.6) | 68(52.3) | .51(.34,.76) | . 45 (.29, .69)** |
| Phone owner |  |  |  |  |
| No | 125(34.9) | 258(66.1) | 1 | 1 |
| Yes | 89(41.5) | 131(58.5) | 1.37(1.1,1.9) | 1.23(.89, 1.8) |
| self-efficacy |  |  |  |  |
| confident | 90(42.05) | 124(57.9) | 1 | 1 |
| Not confident | 124(31.8) | 266(68.2) | .68(.48,.96) | .47 (.69, 1.00) |
| Distance of health center |  |  |  |  |
| Not far | 58(40.5) | 85(59.4) | 1 | 1 |
| far | 156(33.8) | 305(66.2) | .74(.50,.99) | .89(.56, 1.3) |
| ANC follow-up |  |  |  |  |
| No | 76(32) | 161(67.9) | 1 | 1 |
| Yes | 138(37.6) | 229(62.3) | 1.2(.90,1.8) | . 95(.64,1.4) |
| Perceive severity of PIH |  |  |  |  |
| Not sever | 55(26.4) | 153(73.5) |  | 1 |
| sever | 159(40.1) | 237(59.9) | 1.8(1.2,2.6) | 1.6(1.1 , 2.4)* |
| Is PIH a deadly disease? |  |  |  |  |
| Agree | 91(42.9) | 121(57.1) | 1 | 1 |
| Dis agree 123(31.4) | | 269(68.6) | .60(.43,.85) | . 70(.48 ,1.01) |
| Satisfaction of care provider service |  |  |  |  |
| No | 120(30.6) | 273(69.4) | 1 | 1 |
| Yes 94(44.5) | | 117(55.6) | 1.8(1.2,2.5) | 1.7(1.1 , 2.5)* |

364

## Discussion

This study aimed to determine pregnant women's information-seeking behaviors about pregnancy-induced hypertension and the challenges to seeking PIH information in rural Sekela woreda. The findings of this study will be used to determine the feasibility of PIH information sources desired by pregnant women, as well as factors associated with pregnancy-induced hypertension. According to the findings of this study, 35.4% (95% CI = 31%, 39%) of pregnant women sought PIH information from various information sources in the previous 12 months, which is lower than the study conducted in the Tigray region (20). The possible reason might be due to the study participants' residence deference. The study conducted in Tigray was done in a hospital, but the current study is conducted in a rural area. Our findings show that, pregnant women sought PIH information from a variety of health information sources. Similarly, the study conducted in rural Ghana showed that the participants also used different sources. Another reason could be that farmer participants were 13.6% in the previous study but 69.7% in the current study, making it more difficult to find information seeker than in the previous study (64). Regarding to PIH information sources, the current study indicates that 58.1% of pregnant women used healthcare professionals as their first PIH information source. This study is lower than the study conducted in selected hospitals in Ibadan Metropolis; the former study shows that 93.4% of the respondents’ PIH information sources were health care professionals. This deference might be due to the participant’s study area and participant educational statues. In addition to this, the former participants used different sources for their PIH information compared to the current participants. So, this might become a huge difference information sought between participants(65)*.*

In the current study, 51.6% of respondents*'* trusted information sources were healthcare providers. T*he study co*nducted at Florida State University about the source of PIH information seeking, which is consistent with the current study that 58.1% of the participants used health care providers as their PIH information source (66). The study conducted in rural Malaysia states that 58.8% of the participants used their family or friends as their primary source of health information. This study is greater than the current study (32.2%). This deference might be due to family literacy and financial factors prohibiting using other sources like smartphones and magazines(8).

Regarding to Age, we discovered that pregnant women over the age of 35 were less likely to seek PIH information than those under the age of 35. The study conducted in South Korea states that aged women had less intention of seeking health information compared with those who were young aged women (67-69). The possible reason might be that the aged women were not able to access different sources of information, and those women were leave their time with their children, cooking, and other home activities. Furthermore, compared to young women, older women were less literate. A study conducted in the southern provinces of Laos states that aged women did not actively communicate and accept the innovation, so this leads to fewer information seekers compared to those who were younger(18).

The study conducted in Northwest Health Centers of Tehran contradict this study in the former study age is not significant factor for PIH information seeking(32). The possible reason might be the educational level of the study participants. Almost all were diplomas and above. In addition to this, the former study participants were urban, but the current study participants were rural and farmers(32). According to a study conducted in China, most older pregnant mothers were more comfortable in a narrative way of discussing pregnancy-related complications than younger or less than 35-year-old mothers, but in our study, most of the mothers could not read or write, so this finding contradicted our finding ; however, the younger women were more seeker than the elderly (69). The current study was supported by a study conducted at the University of Kebangsaan in Malaysia, which states that the participants who were younger were more information-seekers than the aged participants. The possible reasons might be that the younger participant would be more communicative, technology-accepting, and free-minded compared to the older participant. Another possible reason might be that most of the elderly participants were devoted their time to social life compared to younger participants.

Regarding Family Resistance, it is another significant variable with PIH information seeking. Of the total participants, 21.65% of the pregnant women were resisted by their families. The study conducted in Tamale Metropolis shows that pregnant women were resisted by their families, particularly their husbands, and the majority said that their family did not use medical treatment(14). The current study is similar to the study conducted in India which states that the women faced opposition from their families, especially their husband’s family, if they refused to do household tasks, and not only for the sake of time, but also because they were not interested in seeking information from health facilities (23). The qualitative study conducted India *states that, wome*n were sought deferent information from their husband and mothers, this is due to the shying of mothers to ask professionals and due to “My mother is also a woman and she knows pregnancy best …So, why do we need to go to the doctor for advice ”(12).

The study, which was conducted in low-income countries, states that pregnant women were aware of healthy behaviors and reported practicing them during their pregnancies. The current finding was not similar to the former study because the former study's family were aware of health education as well as diagnosis, but in our study, the family were not aware of health education and diagnosis for the complication or disease of PIH(56).

The qualitative study conducted in Ethiopia states that the health care seeking behavior of pregnant women was influenced during pregnancy, birth, and postpartum by religious and cultural factors(29) So, the former study similarly stated that family resistance was a factor for pregnancy-induced hypertension disorder compared with the current finding. A study conducted at Tanzania, states that when a pregnant woman wants to seek health information related to her pregnancy, the family resists to seeking any pregnancy-related information from health facilities. Instead, they use TBAs and mothers-in-law. This is due to a lack of health care professionals in Tanzania (70).

Regarding the perceived severity of PIH, it is another significant factor for pregnancy-induced hypertension information seeking. The study shows that participants who had perceived severity were 1.6 times more likely to seek PIH information than participants who did not perceive severity of PIH. This study was supported by the study conducted on university students. The former study states that a participant who did not severely perceive the disease did not have the intention of seeking the ways to prevent and as well diagnose the disease or the complication (46). The study conducted at the University of Gondar states that the participants had a high perceived severity, they had high intention of knowing the risk factors, prevention methods, diagnosis, and treatment of the disease(5). Our finding is similar to the study conducted in Dawro zone, Esera woreda, which states that perceived severity is significantly associated with health care seeking information. So, these severely ill participants were more likely to seek healthcare information than those of the participants who were not severely ill(57).

The study conducted in Esera woreda, Ethiopia states that severely ill participant were more information seeker than those who were not severely ill(57).

Regarding to health care satisfaction, it is another significant factor for the outcome variable. Our result shows that, the participantnt who were satisfied by the service of health care provider were more PIH formation seeker than that of the participant who were not satisfied with the service, this finding is similar to The study conducted in Sweden states that, most of the participant were satisfied with the health care provider service were more information seeker than that of the participant who were not satisfied with the service of health care provider(44). The current finding supported by the study conducted in Kano Metropolis, Nigeria states that, a participant who were satisfied with the antenatal care service in the health facility were more information seeker than that of the participant who were not satisfied with the antenatal care service in the facility(71).

**Strength and Limitation of the study**

## Strength of the study

Most of the studies conducted on information seeking on different cases were hospital and organizational based, but this study was conducted in a rural community setting, which is difficult in terms of material feasibility and easy of task accomplishment. As I have seen deferent literature this finding was the first finding in rural area. So, these finding might be used to for reference for the next researchers.

## Limitation of the study

The limitation of the research, it is not including all the rural pregnant women in our sample because of time and resource limitation. The study was a community-based cross-sectional study, which may not articulate the causal inference between variables. The study assessed PIH information-seeking in the past one year. There may have been a chance of recall bias by the participants. The face to face interview was conducted so this leads to missing of information from the respondents because of shying and afraid.

## Conclusion and Recommendation

This study finds a significant proportion of PIH information seeking behavior among pregnant women. The main sources of PIH information for pregnant women were health care professionals and families. But the most trusted information sources were health care providers for pregnant women. In this study, participants sought the information source, types of PIH complications, and also were aware of how the complications might be managed and prevented. The age of the participant, family resistances, Satisfaction of care provider service and perceived severity, were significantly associated with PIH information seeking behavior.

We were expanding health education programs for pregnant women and providing awareness and training about PIH to participants and their husbands is the most effective way to reduce prevalence of PIH complications.

### Acknowledgement

We would like to forward our deepest gratitude to the University of Gondar, Sekela woreda health office, the study participants and the department of Health Informatics.

**Funding**: No funding is available

**Conflict of Interest:** No computing Interest **Authors Contribution:**

Ayana Alebachew: has contribution for the design and conception of the study, collection and analysis of the data, and interpretation of the findings and write the manuscript.

Ayenew Sisay, Sefefe Birhanu , Mulugeta Desalegn, Agmasie Damitew and Adamu Amibachew has also substantial contribution by giving necessary comments from the proposal stage to the final write up of this research and Mr. Kegne Shitu: has substantial contribution in the design and conception of the study and supervise the whole research process during the data collection and write-up.

## Abbreviations

AOR:	Adjusted odds ratio

CI:	Confidence interval

COR: Crude odds ratio

DBP	diastolic Blood Pressure

PIH: Pregnancy Induced Hypertension

SBP: Systolic Blood Pressure

SD:	Standard deviation

WHO:	World health organization

## References

1. Walle TA, Azagew AW. Hypertensive disorder of pregnancy prevalence and associated factors among pregnant women attending ante natal care at Gondar town health Institutions, North West Ethiopia 2017. Pregnancy hypertension. 2019.
2. Kazushi Watanabe1 KN, Kanji Tanaka 3 , Hirohito Metoki4, Yoshikatsu Suzuki 5. Outline of Outline of

Definition	and	Classification	of	“Pregnancy	induced	Hypertension	(PIH)2004.

Availablefrom:https://[www.jstage.jst.go.jp/article/jsshp/1/1/1_3/_pdf.](http://www.jstage.jst.go.jp/article/jsshp/1/1/1_3/_pdf)

3. Belayhun Y, Kassa Y, Mekonnen N, Binu W, Tenga M, Duko B. Determinants of Pregnancy-Induced Hypertension among Mothers Attending Public Hospitals in Wolaita Zone, South Ethiopia: Findings from Unmatched Case-Control Study. International journal of hypertension. 2021.
4. Liu C-M, Cheng P-J, Chang S-D. Maternal complications and perinatal outcomes associated with gestational hypertension and severe preeclampsia in Taiwanese women. Journal of the Formosan Medical Association. 2008.
5. Vahideh Zarea GavganiTabriz University of Medical sciences vyc, Elham QeisariTabriz University of Medical Sciences elyc, Mohammad Asghari JafarabadiTabriz University of Medical Sciences magc. Health Information Seeking Behavior (HISB): A Study of a Developing CountryDeveloping Countr. Reproductive health. 2022;19(4/29/2022).
6. Vahideh Zarea Gavgani (PhD) EQ, Mohammad Asghari Jafarabadi (PhD). Health Information Seeking Behavior (HISB): A Study of a Developing Country.
7. Weaver JB, 3rd, Mays D, Weaver SS, Hopkins GL, Eroglu D, Bernhardt JM. Health Information–Seeking

Behaviors, Health Indicators,and Health Risks. Am J Public Health. 2010;100(8):1520-5.

8. Bakar ABA. Information Seeking Behaviours of Rural Women in Malaysia. 1/1/2011.
9. Ashavaree Das P, and Madhurima Sarkar, PhDb. Pregnancy-Related Health Information-Seeking Behaviors Among Rural Pregnant Women in India: Validating the Wilson Model in the Indian Context.
10. Starling AP, Engel SM, Whitworth KW, Richardson DB, Stuebe AM, Daniels JL, et al. Perfluoroalkyl substances and lipid concentrations in plasma during pregnancy among women in the Norwegian Mother and Child Cohort Study. Environment International. 2014.
11. Singh V, Srivastava M. Associated risk factors with pregnancy-induced hypertension: A hospital-based KAP study. International Journal of Medicine and Public Health. 2015.
12. Ashavaree Das P, aMS, PhDb. Pregnancy-Related Health Information-Seeking Behaviors Among Rural

Pregnant Women in India: Validating the Wilson Model in the Indian Context.

13. Daka FMaK. Maternal health information needs of women: A survey of literature. The Journal of Lexicography and Terminology. Vol 2. Issue 1, 2018;Vol 2. .
14. IMORO WDS. Awareness and health seeking behaviour of pregnant women about pregnancy induced hypertension in the tamale metropolis. 2018.
15. Muti M, Tshimanga M, Notion GT, Bangure D, Chonzi P. Prevalence of pregnancy induced hypertension and pregnancy outcomes among women seeking maternity services in Harare, Zimbabwe. BMC cardiovascular disorders. 2015.
16. *Amah NE, **Alele, I. O. & ***Chukwukere, V. C. Appraisal On Information–Seeking Behaviour Of Rural Women Farmers In Jos South Local Government Area,Plateau State, Nigeria.
17. Shieh C, Mays R, McDaniel A, Yu J. Health literacy and its association with the use of information sources and with barriers to information seeking in clinic-based pregnant women. Health care for women international. 2009.
18. Phommachanh S, Essink DR, Wright PE, Broerse JEW, Mayxay M. Maternal health literacy on mother and child health care: A community cluster survey in two southern provinces in Laos. PloS one. 2021.
19. Jung M. Determinants of health information-seeking behavior: implications for post-treatment cancer patients. Asian Pacific journal of cancer prevention : 2014.
20. Berhe AK, Ilesanmi AO, Aimakhu CO, Bezabih AM. Awareness of pregnancy induced hypertension among pregnant women in Tigray Regional State, Ethiopia. The Pan African medical journal. 2020.
21. Kattah AG, Garovic VD. The management of hypertension in pregnancy. Advances in chronic kidney disease. 2013.
22. James PR, Nelson-Piercy C. Management of hypertension before, during, and after pregnancy. Heart. 2004.
23. Ashavaree Das P, and Madhurima Sarkar, PhDb. Pregnancy-Related Health Information-Seeking Behaviors Among Rural Pregnant Women in india India:.
24. Weldegiorgis SK, Feyisa M. Why Women in Ethiopia Give Birth at Home? A Systematic Review of

Literature. International journal of women's health. 2021

25. sage_idva33_447.pdf.
26. Kaaya ESK, J.Luhanga, E. Maternal knowledge-seeking behavior among pregnant women in Tanzania.

Women's health. 2021.

27. Bakar ABA. Information Seeking Behaviours of Rural Women in Malaysia .pdf.
28. Sudabeh Kamali* LA, Reza Khajouei‡& Kambiz Bahaadinbeigy†.Health information needs of pregnant women information sources, motives and barriers.pdf.
29. Shiferie F, Berhane Y, Workneh F. Healthcare Seeking Behaviour of Pregnant Women in rural Amhara, Ethiopia: Perception of Healthcare Providers. 2020.
30. Ayinadis W. kD, Atsede M. Information seeking on sexually transmitted infections and associated factors among undergraduate students at University of Gondar, Northwest Ethiopia,2021 .
31. Mengiste M, Ahmed MH, Bogale A, Yilma T. Information-Seeking Behavior and Its Associated Factors Among Patients with Diabetes in a Resource-Limited Country: A Cross-Sectional Study. Diabetes, metabolic syndrome and obesity : targets and therapy. 2021.
32. Panahi S, Mahmoudvand F, Sedghi S. Health Information-Seeking Behavior of Iranian first-time Mothers and First-Time Pregnant Women in Northwest Health Centers of Tehran. Journal of Community Health Research. 2020.
33. Debbie Singh MN, Nina Smith and Meg Wiggins. The information needs of first-time pregnant mothers.
34. Jang J, Dworkin J, Hessel H. Mothers' use of information and communication technologies for information seeking. Cyberpsychology, behavior and social networking. 2015.
35. Zamani M, Soleymani MR, Afshar M, Shahrzadi L, Zadeh AH. Information-seeking behavior of cardiovascular disease patients in Isfahan University of Medical Sciences hospitals. Journal of education and health promotion. 2014.
36. Iyalomhe2 GBSIaSI. <health-seeking-behavior-of-rural-dwellers-in-southern-nigeria-implications-forhealthcare-professionals.pdf> Received 27th October 2011
37. MUMBA C. The information- seeking behaviour of women attending antinatal cilinic. 2015.
38. Kamali S, Ahmadian L, Khajouei R, Bahaadinbeigy K. Health information needs of pregnant women: information sources, motives and barriers. Health information and libraries journal. 2018;35(1):24-37.
39. Yilma TM. Health Information Seeking and its Associated Factors among University Students: A Case in a Middle-Income Setting. 2017.
40. Meander L, Lindqvist M, Mogren I, Sandlund J, West CE, Domellof M. Physical activity and sedentary time during pregnancy and associations with maternal and fetal health outcomes: an epidemiological study. BMC pregnancy and childbirth. 2021.
41. Uloma Doris Onuoha AAA. information seeking behaviour of pregnant women in selected hospitals of ibadan metropolis.
42. Nic Newman with Richard Fletcher AS, Simge Andı, Craig T. Robertson, and Rasmus Kleis Nielsen. Reuters Institute Digital News Report 2021 10TH EDITION. 2021.
43. Sjøvaag H, Owren T. The non-substitutability of local news?Advertising and the decline of journalism's umbrella market model. Nordicom Review. 2021.
44. Carlsson M. Cancer patients seeking information from sources outside the health care system. Supportive care in cancer : official journal of the Multinational Association of Supportive Care in Cancer. 2000.
45. Marzieh, Javanmardi1, Mahnaz Noroozi2, Firoozeh Mostafavi3, Ashrafi-rizi4 H. Internet Usage among Pregnant Women for Seeking Health Information: A Review Article.pdf.
46. Gedefaw A, Yilma TM, Endehabtu BF. Information Seeking Behavior About Cancer and Associated Factors Among University Students, Ethiopia: A Cross-Sectional Study. 2020.
47. Kalayou MH, Tilahun B, Endehabtu BF, Nurhussien F, Melese T, Guadie HA. Information Seeking on Covid-19 Pandemic: Care Providers' Experience at the University of Gondar Teaching Hospital, Northwest of

Ethiopia. Journal of multidisciplinary healthcare. 2020.

48. Lambert SD, Loiselle CG. Health Information–Seeking Behavior.pdf.
49. Sutan R, Hassan H, Shamsuddin K. Health Information Seeking Behaviour among Hypertensive Disorder

In Pregnancy (HDP) High Risks Antenatal Mothers. 2016.

50. Dereje S, Yenus H, Amare G, Amare T. Maternity waiting homes utilization and associated factors among childbearing women in rural settings of Finfinnee special zone, central Ethiopia: A community based cross-sectional study. PloS one. 2022
51. Zare-Farashbandi F, Lalazaryan A. A Review of models and theories of health information seeking behavior. International Journal of Health System and Disaster Management. 2014.
52. Merrell LK. exploration of the Pregnancy-Related Health Information Seeking Behavior of Women who Gave Birth in the Past Year.pdf. March 24, 2016.
53. Kassie SY, Melese T, Handebo S, Sebastian Y, Ngusie HS. Information seeking about COVID-19 and associated factors among chronic patients in Bahir Dar city public hospitals, Northwest Ethiopia: a crosssectional study. BMC infectious diseases. 2022.
54. Bayu Begashaw 1, Thomas Tesfaye Healthcare Utilization among Urban and Rural Households in Esera District: Comparative Cross-sectional Study. 2015.
55. Khalid	S	Khan	DW,	Lale	Say,	A	Metin	Gülmezoglu,	Paul	F	A	Van	Look.

WHO_Analysis_of_Causes_of_Maternal_anemi2016

56. Lynne Porter Lewallen P, R.N. Public Health Nursing - 2004 - Lewallen - Healthy Behaviors and Sources of Health Information Among LowLevel Income Pregnant.
57. Bayu Begashaw 1,Thomas Tesfaye 2. Healthcare Utilization among Urban and Rural Households in Esera District: Comparative Cross-sectional Study march, 2015.
58. Ganjali M, Sepehri Z, Amjadi N, Bagheri S, Davoodi M. Knowledge, Attitude and Functioning Toward Pregnancy Induced Hypertension in Pregnant Women Referred to Health Centers in Zabol, 2014.
59. Yilma TM. Health Information Seeking and its AssociatedFactors among University Students: A Case in a Middle-Income Setting.
60. Bitew MS, Zewde MF, Wubetu M, Alemu AA. Consumption of alcohol and binge drinking among pregnant women in Addis Ababa, Ethiopia: Prevalence and determinant factors. PloS one. 2020.
61. Carla S Meurk 1* AB, Jon Adams 3, WHaJL. Factors influencing women’s decisions to drink alcohol during pregnancy: findings of a qualitative study with implications for health communication. 2014.
62. U.S. National Institutes of Health U.S. Department of Health and Human Services , Expiration Date: October 31, 2014.
63. Mohamad Shakir SM, Wong LP, Lim Abdullah K, Adam P. Online STI information seeking behaviour and condom use intentions among young Facebook users in Malaysia. Health Promotion International. 2019.
64. AKPOGHIRAN IP. Communication Source of Seeking Health Information among Women: A Study in Orerokpe, Delta State, Nigeria.pdf. 2020.
65. Amuda UDOAA. Information seeking behaviour of pregnant women in selected hospitals of ibadan metropolis.
66. Ashavaree Das P.Information-Seeking Among Pregnant Women: A Mixed Method Approac. 2013.
67. Noh HIL, J. M.Yun, Y. H.Park, S. Y.Bae, D. S. Nam, J. H.Park, C. T.Cho, C. H.Kye, S. Y.Chang, Y. J.

Cervical cancer patient information-seeking behaviors, information needs, and information sources in South Korea. Supportive care in cancer : official journal of the Multinational Association of Supportive Care in Cancer. 2009.

68. Gudeta TA, Regassa TM. Pregnancy Induced Hypertension and Associated Factors among Women Attending Delivery Service at Mizan-Tepi University Teaching Hospital, Tepi General Hospital and Gebretsadik Shawo Hospital, Southwest, Ethiopia. Ethiopian journal of health sciences. 2019.
69. Liu G, Xue Y, Qian Z, Yang L, Yang Y, Geng Q, et al. Healthcare-seeking behavior among pregnant women in the Chinese hierarchical medical system: a cross-sectional study. International journal for equity in health. 2019.
70. Mwangakala HA. Accessibility of maternal health information and its influence on maternal health preferences in rural Tanzania: A case study of Chamwino District. South African Journal of Information

Management. 2021.

71. Msughter HMNaAE. Survey of Reproductive Health Information Seeking

652	Behaviour among Pregnant Women in Some Selected Hospitals in Kano Metropolis, Nigeria. September 29, 653	2020.

654

655

Supporting Information

[Click here to access/download](https://www2.cloud.editorialmanager.com/pdig/download.aspx?id=45601&guid=07e0781f-011e-4ff6-9cb3-52887cdcf32e&scheme=1)

[Supporting Information table 1.docx](https://www2.cloud.editorialmanager.com/pdig/download.aspx?id=45601&guid=07e0781f-011e-4ff6-9cb3-52887cdcf32e&scheme=1)

Supporting Information

[Click here to access/download](https://www2.cloud.editorialmanager.com/pdig/download.aspx?id=45602&guid=c72464a7-ccaa-4e6d-9029-f66865e115c7&scheme=1)

[Supporting Information image .docx](https://www2.cloud.editorialmanager.com/pdig/download.aspx?id=45602&guid=c72464a7-ccaa-4e6d-9029-f66865e115c7&scheme=1)
