# Supplementary material for: Maternal information-seeking on pregnancy-induced hypertension and associated factors among pregnant women, in low resource country, A cross-sectional study design
Source: PLOS Digit Health. 2025 Mar 18;4(3):e0000740. doi: 10.1371/journal.pdig.0000740 (PMC11957557; doi:10.1371/journal.pdig.0000740)
Supplement: Attachment — Submitted filename: plos_Digital_comment_answer_auresp_2.docx [file pdig.0000740.s008.docx]

| S. no | Reviewer’s comments | Another’s Answer |
| --- | --- | --- |
|  | General comments | Thank you and we received the reviewers comment, this comment is highly interested to increase the quality of the manuscript. We are going to address all the comments and language issues from the editors and the reviewers.  Thank you once again. |
| 1. | Reviewer’s Report for “Maternal  Information-Seeking on Pregnancy-Induced  Hypertension and Associated Factors among  Pregnant Women in a Low-Resource Country: A Cross-Sectional Study”  November 18, 2024 | Summary  This manuscript addresses the critical challenge of pregnancy-induced hypertension (PIH), a significant medical condition affecting 6–10% of pregnancies globally and posing severe risks to maternal health. The lack of information-seeking  behavior regarding PIH among expectant mothers exacerbates the likelihood of  Complications and maternal mortality. This study investigates the information seeking behaviors related to PIH and associated factors among pregnant women in the rural Sekela district. The findings reveal that PIH-related information seeking is generally low, with healthcare providers identified as the primary source of information. Factors influencing information-seeking include maternal age, family resistance, satisfaction with healthcare services, and perceived severity of PIH. |
| 2. | 2 Comments  • Proofreading: The manuscript requires thorough proofreading, particularly in the introduction and conclusion sections. While these sections provide a good overview, the lack of clarity and grammatical issues may hinder comprehension from a general reader’s perspective.  • Visualization of Data: The manuscript includes several tables. To  enhance clarity and reader engagement, consider representing the data  as percentage-stacked bar plots or other suitable visualizations for better interpretation.  1  • Clarity in Methodology: The methods section needs clearer expla-  nations, particularly regarding the binary and multivariable logistic re-  gression techniques used in the analysis. The current description may be  Difficult for readers to follow.  3 Overall Recommendation  I recommend that the paper be revised to address the aforementioned issues  before further consideration for publication. | We would like to address our gratitude thanks for the reviewer to overlook the quality of manuscripts.  We have checked the manuscript grammar, spelling and other inappropriate contents added in our manuscript.  The visualization of our finding is now clearly addressed for the readers.as we have seen in the main document our tales were put under the description. This makes that when readers see this manuscript, there is clear discussion for the table. When we were increase the tale due to increase simply understanding by readers. When we see each tables there is clearly discussion, this increases easily understand.  Thank you for the issues razed and we have corrected in the main document  We were used binary logistic regression for checking dependent variables with independent variables, during this time the p-value is less than or equals to 0.02, we are going to use for multivariable logistic regression to identify which independent variable is highly risk factors for the outcome variables which is PIH information seeking. For readers clarity we were clearly wrote in the main document and it is really clear for readers.    Thank you this considerations , this idea is used for the increment of the quality of the paper for publication, |
