# Supplementary material for: Maternal information-seeking on pregnancy-induced hypertension and associated factors among pregnant women, in low resource country, A cross-sectional study design
Source: PLOS Digit Health. 2025 Mar 18;4(3):e0000740. doi: 10.1371/journal.pdig.0000740 (PMC11957557; doi:10.1371/journal.pdig.0000740)
Supplement: Attachment — Submitted filename: plos Digital comment answer.docx [file pdig.0000740.s006.docx]

| S. no | Reviewer A: comments | Another’s Answer |
| --- | --- | --- |
|  | General comments | Thank you and we received the reviewers comment corrected the issue raised |
| 1. | Reviewer #1: Thank you for your nice work, the topic is interesting, and this study can add value to the concept of perinatal health.  I would like to mention to some point you need to address:  • In the result section of the abstract, line 37, you mentioned that the proportion of PIH information seeking among pregnant women was 214?? 214 out of what, either right the total number or keep only the percentage | Thank you for your deep insight, We were corrected the comment from the main document. From 604 participants 214 were PIH information seeker. We were include the number due to clarity of the readers. |
|  | Typo in line 80.  • I think the sentence in lines 89, 90 is in complete, what is the relationship??  • Typo in line 130. | Thank you for the comment you raised and We have seen and incorporate in the main document.  Health literacy refers to the ability of individuals to obtain, process, and understand basic health information and services needed to make informed health decisions.  So, if there is health literacy there is also the information about PIH |
|  | Where you interviewed or you did interview pregnant women, line 152 | We were held the interview at Sekela woreda and we were include at the main document |
|  | Review line 167.  Typo in 210 | We have corrected based on the comment in the main document |
| 2. | Reviewer #2: | |
|  | The paper explores the impact of digital health interventions on managing Pregnancy-Induced Hypertension (PIH) in rural Ethiopia. To strengthen its scientific impact, consider addressing the following aspects:  1. Originality and Novelty:  o Clearly highlight the novel aspects of your research and how it differs from existing studies. A comparative analysis with recent similar research can help establish the unique contributions of your work.  2. Importance and Broad Interest:  o Emphasize the broader implications of your findings. Articulate how your study influences practice, policy, or further research in digital health, and how it could be relevant beyond the specific context of rural Ethiopia.  3. Methodological Rigor and Ethical Standards:  o Provide a more detailed description of your study design, data collection methods, and analysis techniques. Ensure all ethical considerations are thoroughly addressed, including obtaining necessary approvals.  4. Substantial Evidence:  o Include additional analyses or data to further substantiate your conclusions. Address any study limitations openly and discuss how future research could address these limitations.  5. Utility and Accessibility:  o Elaborate on the practical applications of your findings. Ensure that the implications for practice and policy are clear and accessible to a wide audience, including practitioners and policymakers.  6. Open Science Standards:  o Deposit your data and related protocols in public repositories to enhance transparency and reproducibility. Provide links or access information in your manuscript.  7. Reproducibility and Protocols:  o Detail your methodological protocols to facilitate replication of your study. If there were deviations from standard practices, explain and justify these deviations clearly.  8. Clear and Accessible Writing:  o Revise the manuscript to improve clarity and coherence. Ensure that complex concepts are explained in an accessible manner for non-specialists.  9. Supporting Information:  o Consider including detailed protocols, algorithms, or additional data as supplementary materials. This can enhance the transparency and depth of your research.  10. Compliance with Guidelines:  o Confirm and mention adherence to relevant guidelines (e.g., CONSORT, STROBE) to ensure the methodological rigor of your study. | Thank you for the comments: we were incorporate you’re your comments in the main document. When we see the originality of the manuscript, this manuscript is original and followed the research guideline and real data results that is collected from really form the participants so, this paper is original.  The novelty of this paper is identifying the seeking behavior of PIH among pregnant mothers and this manuscript identified patterns of how much mothers were seek and did not seek PIH related disease easily.  The importance and implication of this manuscript is in Ethiopia ministry of health has a slogan which is “no mothers die due to delivery” so this paper leads to plan and solve problem that leads to maternity problems like lack of awareness of pregnancy induced hypertension related disease and what are the factors that occurs during pregnancy, so this manuscript is easily identifying the factors. The manuscript recommended the health care professionals to give proper health education for mothers and this manuscript also recommended the health police to give reproductive health education for not only form mothers but also house hold heads for farther awareness of PIH related disease  Methodological Rigor and Ethical Standards:  Thank you for the comment and we were wrote followed clear and standardized methodology to identify the factors that mothers leads to pregnancy induced problem we were report at the result section. There is study design study population sampling method and sampling procuders.  Regarding to Ethical consideration: we have had ethical clearance from the university of Gondar institute of public health IRB.  Regarding to the guidelines we have used STROBE (Strengthening the Reporting of Observational Studies in Epidemiology) we have used cross sectional study design to identify current factors to affect related to PIH, and we have wrote the results and recommended for concerned body based on our results. |
| 3 | Reviewer #3: | |
| 1. | The use of English is below average and requires professional proof reading to meet international standard.  The scope of this study seems not to align with digital health in anyway, I would suggest the author consider submission to pure public health or health communication journal | Thank you for your insight and we have proof language problem. Thank you for your recommendation of other journals after the decision of this journal we will try to submitted to this journal. |
